# Effects of High and Low Protein Diets on Inflammatory Profiles in People with Morbid Obesity: A 3-Week Intervention Study

**DOI:** 10.3390/nu12123636

**Published:** 2020-11-26

**Authors:** Liselot Koelman, Mariya Markova, Nicole Seebeck, Silke Hornemann, Anke Rosenthal, Volker Lange, Olga Pivovarova-Ramich, Krasimira Aleksandrova

**Affiliations:** 1 Senior Scientist Group Nutrition, Immunity and Metabolism, Department of Nutrition and Gerontology, German Institute of Human Nutrition Potsdam-Rehbruecke, 14558 Nuthetal, Germany; liselot.koelman@dife.de; 2 Institute of Nutritional Science, University of Potsdam, 14558 Nuthetal, Germany; seebeck.ni@gmail.com; 3 Department of Clinical Nutrition, German Institute of Human Nutrition Potsdam-Rehbruecke, 14558 Nuthetal, Germany; maria.markova@yahoo.de (M.M.); silkehornemann@o2mail.de (S.H.); olga.ramich@dife.de (O.P.-R.); 4 German Center for Diabetes Research (DZD), 85764 Munich-Neuherberg, Germany; 5 Clinic for Nutritional Medicine, 10625 Berlin, Germany; Anke@dr-rosenthal.com; 6 Center for Obesity and Metabolic Surgery, Vivantes Hospital, 13585 Berlin, Germany; Volker.Lange2@helios-gesundheit.de; 7 Helios Klinikum Berlin-Buch, 13125 Berlin, Germany; 8 Department of Endocrinology, Diabetes and Nutrition, Charitè University Medicine Berlin, Campus Benjamin Franklin, 12200 Berlin, Germany; 9 Research Group Molecular Nutritional Medicine, Department of Molecular Toxicology, German Institute of Human Nutrition Potsdam-Rehbruecke, 14558 Nuthetal, Germany; 10 Department Epidemiological Methods and Etiological Research, Leibniz Institute for Prevention Research and Epidemiology-BIPS, 28359 Bremen, Germany

**Keywords:** high protein, low protein, nutritional intervention, morbid obesity, inflammatory biomarkers

## Abstract

Nutritional interventions in morbidly obese individuals that effectively reverse a pro-inflammatory state and prevent obesity-associated medical complications are highly warranted. Our aim was to evaluate the effect of high (HP) or low (LP) protein diets on circulating immune-inflammatory biomarkers, including C-reactive protein (CRP), interleukin-6 (IL-6), tumor necrosis factor-alpha (TNF-a), interleukin-10 (IL-10), monocyte chemoattractant protein-1 (MCP-1), chemerin, omentin, leptin, total adiponectin, high molecular weight adiponectin, and fetuin-A. With this aim, 18 people with morbid obesity were matched into two hypocaloric groups: HP (30E% protein, *n* = 8) and LP (10E% protein, *n* = 10) for three weeks. Biomarkers were measured pre and post intervention and linear mixed-effects models were used to investigate differences. Consuming HP or LP diets resulted in reduced CRP (HP: −2.2 ± 1.0 mg/L, LP: −2.3 ± 0.9 mg/L) and chemerin (HP: −17.9 ± 8.6 ng/mL, LP: −20.0 ± 7.4 ng/mL), with no statistically significant differences by diet arm. Participants following the LP diet showed a more pronounced decrease in leptin (−19.2 ± 6.0 ng/mL) and IL-6 (−0.4 ± 0.1 pg/mL) and an increase in total adiponectin (1.6 ± 0.6 µg/mL). Changes were also observed for the remaining biomarkers to a smaller degree by the HP than the LP hypocaloric diet, suggesting that a LP hypocaloric diet modulates a wider range of immune inflammatory biomarkers in morbidly obese individuals.

## 1. Introduction

The worldwide prevalence of obesity has nearly tripled since 1975 [1]. As the prevalence of obesity has increased globally, adverse health risks and healthcare expenditure have amplified at an accelerating rate [2]. Especially worrying is the increasing proportion of people with morbid obesity characterized by body mass index (BMI) ≥ 35 kg/m^2^ [3]. These people are exposed to a higher risk of various chronic diseases, premature ageing, and overall mortality. Along with metabolic complications such as hyperinsulinemia, hypertension, and hyperlipidemia, obesity also leads to a disturbed immune balance and chronic low-grade inflammation [4].

Excess adipose tissue provides an environment for secretion of multiple cytokines and hormones that exert regulatory functions in energy metabolism, inflammation, and insulin sensitivity [5]. In morbid obesity, the immune system is especially challenged and constantly struggles to cope with the flow of these proinflammatory triggers and preserve healthy functioning of all organs and systems [6,7]. Finding approaches to lower obesity may also support the immune system in its battle with the systemic pro-inflammatory response and favorably influence overall health. General lifestyle interventions such as low calorie diets and physical activity regimens have been shown to have low compliance and limited effectiveness in people with severe obesity [8]. Bariatric surgery has gained increasing popularity as a treatment strategy in patients with morbid obesity [9]. Patients that have undergone bariatric surgery experience lower inflammatory concentrations and improved insulin resistance that could be explained by reduced systemic and adipocyte inflammation and secretion of adipocyte derived cytokines [10,11]. However, both surgical treatment and weight loss interventions have not proven successful in the long run [8,12,13]. The challenge remains to identify novel strategies that bear potential for obesity treatment and management in people with severe obesity targeted at specific pathophysiological pathways.

Emerging evidence shows that dietary components can modulate key pathways to inflammation. For instance, omega-3 fatty acid intake can dampen NF-kB activation and modulate the magnitude of inflammatory responses to stressors [14]. Dietary flavonoids have also been found to be capable of modulating cytokines and C-reactive protein (CRP) production in intervention studies [15]. However, for individuals with morbid obesity, adapting to diets that consist of specific food components may be a challenge. In this vein, dietary plans balancing macronutrient composition may represent a promising and non-drastic intervention approach that can be adopted by people with morbid obesity in sustaining long-term health goals.

Over the recent years, evidence emerged to suggest that high protein diets may have beneficial effects on postprandial and fasting glucose concentrations [16], postprandial satiety [17], as well as on blood pressure and blood lipids [18]. High protein diets were particularly suggested to modulate inflammatory concentrations in patients with obesity and diabetes [19] and in the ageing population [20,21]. On the other hand, a low protein diet, especially low methionine diet, was shown to beneficially influence glucose intolerance [22] and modulate an immune-inflammatory state [23,24].

Overall, evidence on the role of both high and low protein diets in modulating metabolic and inflammatory profile in individuals with obesity has been increasing over recent years, hence it remains inconclusive. No studies have simultaneously assessed the effects of high and low protein diets on inflammatory profiles captured by multiple biomarkers. This may be particularly important because single biomarkers may not sufficiently capture the effect of diet on the complete inflammatory phenotype associated with obesity [25]. Studies in people with morbid obesity that may benefit most from such interventions are particularly sparse [13].

To address these gaps, we aimed to evaluate the effect of a 3 week low protein (LP) and a high protein (HP) hypocaloric dietary intervention on immune-inflammatory profiles depicted by various serum biomarkers measured in individuals with morbid obesity.

## 2. Materials and Methods

### 2.1. Study Design and Dietary Intervention

**Figure 1:**
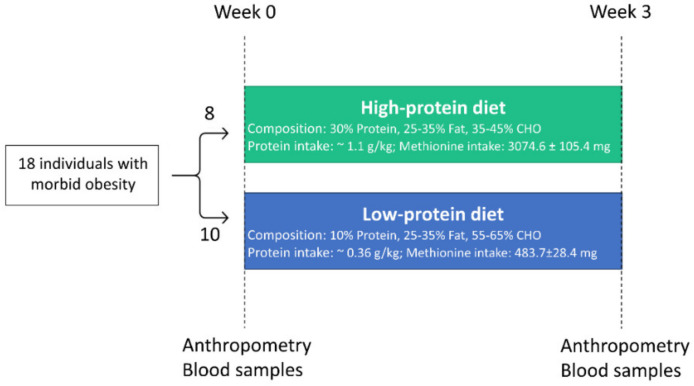
Study design of the intervention study. In total, *n* = 18 participants completed the intervention study and were included for the final analysis. Participants were matched according to age, sex, and body mass index into high protein and low protein diet groups. Single blood samples and anthropometric measurements were collected on two occasions: before the intervention and after 3 weeks.

We used data collected from a dietary intervention study that included 20 patients with morbid obesity (*n* = 7 males and *n* = 13 females) aged 40–50 years old who were recruited from patient lists of the Vivantes Klinikum, Berlin, Germany in the period between January 2016 and June 2017. The primary objective of the original study was to investigate whether LP or HP diets exert greater effects on liver fat reduction [26]. A secondary objective of the study was to assess the effect of high and low protein diets on changes in inflammatory biomarkers. Inclusion criteria were people with BMI > 40 kg/m^2^ or BMI > 35 kg/m^2^ and obesity related co-morbidities (type 2 diabetes, hypertension, dyslipidemia, obstructive sleep apnoe syndrome). Patients were excluded if they were suffering from liver cirrhosis, infectious disease, cancer, or interfering chronic diseases. Participants were randomized into two intervention groups, but due to unsuccessful randomization, they were matched for age, sex, and body mass index (BMI). The nutrient composition of LP and HP diets are presented in Table S1. Participants received either a hypocaloric (1500–1600 kcal/day) high protein (HP: 30 E% protein, 25–30 E% fat, 35–45 E% carbohydrates, *n* = 10) or low protein (LP: 10 E% protein, 25–35 E% fat, 55–65 E% carbohydrates, *n* = 10) diet for three weeks. *n* = 2 participants were excluded due to insufficient repeated biomarker measurements and non-compliance of a high protein diet, measured by reduction of serum urea. This resulted in 18 participants who completed this study (*n* = 7 males, *n* = 11 females) (see Figure 1 and Figure S1).

The HP diet consisted of 3074.6 ± 105.4 mg methionine, whereas the LP diet included 483.7 ± 28.4 mg methionine. Participants received food plans with 10 d rotating menus including recipes. HP food plans consisted of low fat dairy products, eggs, meat, fruits, and vegetables, whereas LP food plans consisted mainly of bread, rice, potatoes, soy products, fruits, and vegetables. Sweets, soft drinks, and cookies were excluded from diets in both groups. There were two follow-up phone calls which took place after week 1 and week 2 of the intervention. Exemplary food plans for both intervention groups can be found in Table S3. Part of the food was provided to the participants (e.g., protein shake including 42% calcium caseinate, 40% soy protein isolate, and 17% whey protein; produced by WellMix Sport Protein 90, WellMix, Burgwedel, Germany). Blinding of the participants was not possible due to providing them with food plans and complementary foods. Food protocols were made with the help of PRODI (Nutri-Science GmbH, Hausauch, Germany).

At the beginning (week 0) and at the end of the intervention (week 3), anthropometric measurements (weight, height, and waist and hip circumference), fasting blood sample collection, and body composition determination via BOD POD (Cosmed, Rome, Italy) were performed. Compliance to the dietary interventions was assessed based on measurements of serum urea as a biomarker of protein intake. In support of compliance to the low protein diet, the levels of urea decreased substantially in the LP group (*p* < 0.05). Vice versa, the compliance to the high protein diet intervention was supported by increases in urea levels in the HP group (*p* < 0.05).

The trial was approved by the Ethics Committee of the Charité University Medicine in Berlin (Application No. EA4/006/15), conducted in accordance with the Declaration of Helsinki. All participants provided written informed consent before entering the study.

### 2.2. Biomarker Measurements

The following biomarkers were measured to assess inflammatory profiles in study participants: C-reactive protein (CRP), interleukin-6 (IL-6), tumor necrosis factor alpha (TNF-a), interleukin-10 (IL-10), monocyte chemoattractant protein 1 (MCP-1), chemerin, omentin, leptin, total adiponectin, high molecular weight (HMW) adiponectin, and fetuin-A. Non-HMW adiponectin was estimated based on the difference between total and HMW adiponectin. Venous blood samples were immediately centrifuged and frozen at −80 °C until analysis. CRP concentrations were determined by a highly sensitive immunoturbidimetric assay using ABX Pentra 400 reagents on an ABX Pentra 400 (Horiba ABX, Montpellier, France). Commercially available ELISA kits were used for the measurements of serum leptin (R&D Systems, Minneapolis, MI, USA), total adiponectin, chemerin, omentin, fetuin-A (all from Biovendor, Germany), and high molecular weight adiponectin (Merck Millipore KGaA, Darmstadt, Germany) and U-Plex assay was used to measure IL-6, TNF-a, MCP-1, and IL-10 (MSD, Rockville, ML, USA). Table S4 provides the detection limits of the kits for the measured biomarkers.

### 2.3. Statistics

Descriptive characteristics presented as medians and interquartile ranges were calculated for all study participants at study baseline. Associations among immune-inflammatory biomarker measurements at baseline were explored using Spearman partial correlation coefficients adjusted for age, sex, and BMI. Corresponding *p* values and 95% confidence intervals were calculated using Fisher’s z transformation.

To evaluate the effect of low protein (LP) and high protein (HP) diets on serum concentrations of immune-inflammatory biomarkers, the differences in outcome variables between baseline and post-intervention were calculated using linear mixed-effects models with a restricted maximum likelihood (REML) method. The fixed effects were modelled for intervention to get the between-subject effect and for time to get the within-subject effect. The random effects were the individual participants. We evaluated the group-by-time interaction to assess the extent to which there are differences between groups over time. We also evaluated the time effect, which shows the effect of energy restriction over time independent of diet. In order to make pairwise comparisons of biomarkers per diet group over time, we computed differences of least squares means where the obtained *p* value was based on the *t*-test. The models were adjusted for age, sex, BMI change, and baseline biomarker measurement in order to correct for weight changes over time and differences in baseline values. Kenward-Roger correction was applied for analysis of mixed models [27]; an approach based on estimated covariance parameters in formulas that assume these are known. This corrects for naive test statistics biased upward and standard errors biased downwards.

All statistical analyses were performed using SAS software package, release 14.2 (SAS Institute, Cary, NC, USA). A figure illustrating differences in least squares means of biomarkers was created in R Studio with ggplot2 package. *p* values were obtained from *t*-test and are two-sided.

## 3. Results

In total, 18 participants completed the intervention with repeated biomarker measurements. Table 1 shows the baseline characteristics of the study population. The LP group consisted of 10 participants (6 females/4 males) with a median age of 48.7 (38.1–56.0) years, median weight at baseline of 126.8 (117.0–157.1) kg, and median BMI at baseline of 43.5 (43.1–47.4) kg/m^2^. The HP group consisted of 8 participants (5 females/3 males), with a median age of 48.4 (44.9–55.7) years, median weight at baseline of 154.2 (121.4–160.4) kg, and median BMI at baseline of 45.1 (42.3–47.9) kg/m^2^. Overall, there were no differences in pre-existing comorbidities between the two groups of participants (data not shown).

Table S2 presents the correlations among the evaluated biomarkers at baseline, adjusted for age, sex, and BMI at baseline. IL-6 correlated positively with CRP (ρ: 0.71; 95% CI: 0.28–0.89) and leptin (ρ: 0.64; 95% CI: 0.16–0.86), whereas inverse associations were seen for omentin with MCP1 (ρ: −0.54; 95% CI: −0.82–−0.02) and fetuin-A with IL-10 (ρ: −0.57; 95% CI: −0.81–0.01).

Table 2 presents the baseline and post-intervention median biomarker concentrations, the time effects, and group-by-time interactions. For biomarkers CRP, IL-6, chemerin, leptin, and total adiponectin, we found significant effects over time independent of diet. No group-by-time interaction effect could be detected for the measured biomarkers. Following the intervention, participants in both groups lost a similar amount of weight (estimate difference (standard error): −4.6 (1.1), *p* < 0.05 for the HP diet and −5.3 (1.0), *p* < 0.0001 for the LP diet; *p*-difference between diets = 0.662). The corresponding decrease in BMI was −1.5 (0.3), *p* < 0.05 for the HP diet and −1.7 (0.3), *p* < 0.0001 for the LP diet, respectively (*p*-difference between diets = 0.377).

**Figure 2:**
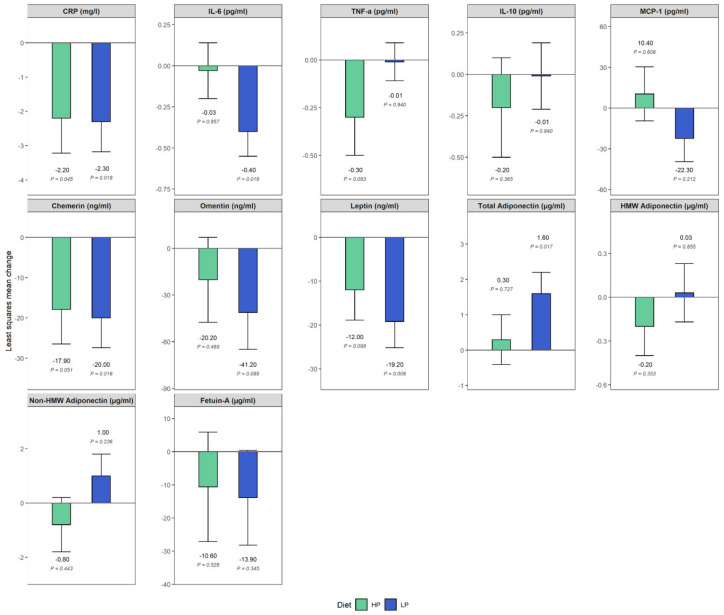
Differences of least squares means of biomarkers over time, grouped by diet, adjusted for age, sex, body mass index change, and baseline biomarker values. Participants receiving a high protein (HP) diet are represented in green; participants receiving a low protein (LP) diet are represented in blue. *p* values are obtained from *t*-test. Abbreviations: CRP, C-reactive protein; IL-6, interleukin-6; TNF-a, tumor necrosis factor alpha; IL-10, interleukin-10; MCP-1, monocyte chemoattractant protein 1; HMW, high molecular weight.

Figure 2 shows the estimated differences of least squares means of biomarkers over time per diet group, adjusted for age, sex, BMI change, and baseline biomarker values. Following either HP and LP diet resulted in reduced concentrations of CRP and chemerin in both intervention arms (CRP: estimate ± SE in HP and LP: −2.2 ± 1.0 mg/L; *P*-diff: 0.045 and −2.3 ± 0.9 mg/L; *P*-diff: 0.019 and chemerin: −17.9 ± 8.6 ng/mL; *P*-diff: 0.051 and −20.0 ± 7.4 ng/mL; *P*-diff: 0.016, respectively). Further, following the LP diet resulted in a reduction in concentrations of IL-6 and leptin (IL-6: −0.4 ± 0.1 pg/mL; *P*-diff: 0.018 and leptin: −19.2 ± 6.0 ng/mL; *P*-diff: 0.006, respectively); whereas total adiponectin concentrations increased (1.6 ± 0.6 µg/mL; *P*-diff: 0.017). Changes in concentrations, albeit less pronounced, were further observed for the following biomarkers: omentin, HP, and LP: −20.2 ± 27.3 ng/mL; *P*-diff: 0.469 and −41.2 ± 23.6 ng/mL; *P*-diff: 0.099; fetuin A, HP, and LP: −10.6 ± 16.5 µg/mL; *P*-diff: 0.528 and −13.9 ± 14.3 µg/mL; *P*-diff: 0.345; TNF-a, HP, and LP: −0.3 ± 0.2 pg/mL; *P*-diff: 0.083 and −0.01 ± 0.1 pg/mL; *P*-diff: 0.940; and leptin, HP: −12.0 ± 6.9 ng/mL; *P*-diff: 0.098.

## 4. Discussion

In this dietary intervention study, we aimed to study the effect of hypocaloric HP and LP diets on immune-inflammatory biomarkers over three weeks. We found that adherence to either a hypocaloric HP or LP diet resulted in reduced concentrations of various inflammatory biomarkers in people with morbid obesity. Results were especially pronounced for CRP and chemerin, two biomarkers reflecting inflammation and cardiovascular risk. Following a LP diet was also associated with a more pronounced decrease in leptin and IL-6 concentrations and an increase in adiponectin concentrations. Effects were less prominent for the remaining biomarkers. To our knowledge, this is the first intervention study that explored the effects of varying amounts of dietary protein on changes in various immune-inflammatory biomarkers in people with morbid obesity.

Our results suggested that the LP diet may be associated with a wider range of beneficial effects, including reducing concentrations of CRP, IL-6, chemerin, and leptin and increasing total adiponectin concentration, whereas the effect of the HP diet seems to be most pronounced in reducing CRP and chemerin concentrations. However, due to the small sample size and the lack of significant group*time interaction, these results should be interpreted with caution and require confirmation in further research. A LP diet can also be characterized by reduced exposure to methionine and a number of animal studies have shown that methionine restriction modulates metabolism and improves health span [28,29]. Low methionine diets have been shown to decrease inflammation [28,30,31], reduce adiposity [32,33], decrease oxidative stress [34], and increase insulin sensitivity [32,35,36]. Compared to calorie restriction, responses to methionine restriction were found to be more robust over the long-run [31]. Dietary methionine restriction has been especially associated with metabolic changes in adipose tissue and the liver, resulting in enhanced insulin sensitivity and energy expenditure [37]. In animal studies, methionine restriction was shown to reduce concentrations of insulin, insulin-like growth factor-1, glucose, and leptin and increased adiponectin [37]. However, evidence from human research has been sparse. In a large cross-sectional study of US adults, methionine-rich diets were associated with a higher prevalence of cardiometabolic disease risk factors, i.e., higher levels of cholesterol, glucose, glycated hemoglobin, uric acid, and insulin [37]. The concentrations of CRP were also higher with a higher intake of methionine-rich diet, albeit the trend did not reach statistical significance. A randomized trial that evaluated the effect of a 16-week methionine restricted intervention (> 80% relative to controls) showed that people with obesity and metabolic syndrome had increased adiponectin concentrations [38]. As our participants in the LP group received both a calorie restricted diet and reduced methionine, a next step would be to reproduce the beneficial effects of the methionine restricted diet in people with morbid obesity without imposition of severe calorie restriction.

To compensate for the reduced protein content, the LP group received a higher proportion of carbohydrates, whilst proportion of fat remained the same in both diets. Accumulating evidence suggests a mediating role of dietary fiber on pro-inflammatory processes by either decreasing oxidation of glucose and lipids while maintaining a healthy gut environment or by altering adipocytes and cytokines in adipose tissue and increasing circulation of lipids and lipophilic compounds [39]. Several studies have found a link between a high fiber diet and reduced plasma CRP, IL-6, and TNF-a [40], so the effects seen in the LP group may also be explained by a change in carbohydrate content. Our findings for CRP are in line with a previous study where CRP was reduced more strongly in adults receiving a LP diet compared to a HP diet [24].

The beneficial effects of HP were restricted to reducing concentrations of CRP and chemerin. These results are in line with our previous work where we evaluated the effect of HP diet in a 6-week intervention study among diabetes patients with obesity [25]. HP has a stronger effect on satiety compared to diets of LP content and with equivalent quantities of E from carbohydrate or fat [41]. Although there is no formal definition of “high protein” as percentage of E in a diet, above 25% E can be seen as high based on a review on satiety and US dietary recommended intakes [42]. The effects seen in HP diets may be explained by the high protein content per se, however they may also be confounded by other components in the diet or the source of the protein. Based on studies assessing the effects of several types of protein, lower consumption of protein from red meat and higher consumption of proteins from vegetables and dairy may decrease a pro-inflammatory state [43,44]. The HP diet in this study and in our previous study contained dairy components. In particular, fermented dairy products (i.e., yoghurt) have been associated with lower levels of inflammation in observational and intervention studies [45,46]. These anti-inflammatory effects could possibly be accounted for by beneficial properties of bacteria species [47] and bioactive peptides that interact with gut microbes and immune cells [48]. Further work would be warranted to explore the influence of dietary interventions on gut microbiota composition and immune status in people with morbid obesity.

Up to date, there is still no consensus as to which biomarkers may best represent low-grade inflammation [49]. Most dietary intervention studies have been limited in the range of evaluated inflammatory biomarkers [15]. CRP is the most established biomarker of inflammation, often used as proxy, sometimes together with IL-6, which stimulates production of CRP. However, CRP alone may not sufficiently capture the effect of diet on the complete inflammatory phenotype associated with obesity. We therefore assessed additional circulating molecules that have been suggested as biomarkers of increased risk and contributing to the pathophysiology of comorbidities of obesity. We were especially interested in evaluating established adipokines such as adiponectin and leptin, as well as novel proinflammatory adipokines, i.e., omentin, chemerin, and MCP-1 were shown to induce insulin resistance, endothelial dysfunction, and systemic inflammation [50]. We were further interested in specific immune-related biomarkers, i.e., chemokines and cytokines that mediate both immune cell recruitment and complex intracellular signaling control mechanisms in obesity, inflammation, and chronic disease development [51]. Finally, we focused on fetuin-A as a biomarker of fatty liver and inflammation, which is known to exert important roles in the pathophysiology of insulin resistance and atherosclerosis [52].

This study also has several limitations. We used data from a clinical trial that was designed and powered to study the effects of LP and HP diets on changes in liver fat, whereas the outcome of our study was changes in inflammatory biomarkers. The sample size was relatively small, which could have influenced the precision of the observed results. In addition, the duration of the intervention was short, so how long the effects of the intervention will last and whether similar effects will be seen in the long run is yet to be revealed. Another factor to consider is the different composition of fat between the HP and LP diets. Although the total fat % of the diets was similar, the amount of saturated fatty acids (SFA) was higher in the HP diet (50% of total fat content) as compared to the LP group (25% of total fat content). SFAs have been reported to affect the immune system and promote inflammation [53] in mice and humans. Therefore, the content of SFA may have been masking the effect of the HP and LP diets on inflammatory biomarkers such that more pronounced anti-inflammatory effects could be seen for the LP diet due to a lower SFA content and less pronounced anti-inflammatory effects could be observed in the HP diets due to a higher SFA content. Furthermore, the intervention consisted of a hypocaloric diet, so participants lost weight. The caloric restriction of these patients may have acted as an activator of protective metabolic pathways, in addition to protein intake or methionine restriction. In the analysis, we adjusted for BMI change pre and post intervention, however the molecular mechanisms underlying the effects of dietary protein or the metabolic effects of weight change may not have been captured sufficiently by the adjustment of BMI. We conducted this study to see whether a change in protein content or methionine restriction in terms of the hypocaloric diet could improve inflammation. If the participants maintained their usual caloric intake, the effects of protein or methionine per se would have been captured better. As there are a number of modifying factors that affect the concentration of an inflammatory marker at a given time [54], including age, diet, and body fatness, among others, we controlled (diet) or corrected (age, sex, BMI) for these in our analyses.

## 5. Conclusions

In this intervention study, adherence to either a HP or LP diet effectively modulated concentrations of inflammatory biomarkers in individuals with morbid obesity. These effects were more pronounced for the LP diet which led to modulation of a wider range of inflammatory targets, including the adipokines leptin and adiponectin. Further studies with a larger size and duration, as well as encompassing a wider range of obesity categories, would be warranted to evaluate the role of high and low protein diets in modulating inflammatory profiles in obesity.

## Figures and Tables

**Table 1:** Descriptive characteristics of study population, according to diet.

Characteristics	High Protein (*n* = 8)	Low Protein (*n* = 10)	*p*-Value
**Demographics**			
Age (years)	48.4 (44.9–55.7)	48.7 (38.1–56.0)	0.859
Female-*n* (%)	5 (62.5)	6 (60.0)	0.916
**Anthropometrics**			
Weight (kg)	154.2 (121.4–160.4)	126.8 (117.0–157.1)	0.424
Waist circumference (cm)	135.0 (123.1–150.8)	134.5 (124.3–145.0)	0.756
Waist-to-hip ratio	0.9 (0.9–1.1)	0.9 (0.9–1.1)	0.688
Body mass index (kg/m^2^)	45.1 (42.3–47.9)	43.5 (43.1–47.4)	0.722
Fat mass (%)	55.4 (51.4–61.1)	54.4 (50.8–56.2)	0.643

Data are shown as median (interquartile range). Abbreviations: cm, centimeters; kg, kilograms; m, meters; *n*, number. *p* values are based on Kruskal-Wallis and Chi-Square test.

**Table 2:** High and low protein diet intervention effects on circulating immune-inflammatory biomarkers.

	Assessment Period				Time Effect ß (95% CI) ^1^	Group-by-Time Interaction ß (95% CI) ^1^
	Baseline		Week 3		Ref: Baseline	Ref: LP*Baseline
	Median (95% CI)	*n*	Median (95% CI)	*n*		
**CRP (mg/L)**						
HP	10.0 (3.9–16.2)	8	3.5 (2.5–11.8)	7	−2.3 (−4.1, −0.4)	0.08 (−2.7–2.9)
LP	10.5 (4.1–14.2)	10	6.4 (2.7–11.4)	10		
*p* value					0.019	0.955
**IL-6 (pg/mL)**						
HP	1.7 (0.8–2.3)	8	1.4 (0.9–2.1)	7	−0.4 (−0.7, −0.08)	0.4 (−0.1–0.8)
LP	2.3 (1.4–3.2)	10	1.8 (1.1–2.8)	10		
*p* value					0.018	0.130
**TNF-a (pg/mL)**						
HP	2.5 (2.3–2.6)	8	2.3 (1.8–2.7)	7	−0.0 (−0.3, 0.3)	−0.3 (−0.8–0.2)
LP	3.1 (2.4–3.4)	10	3.0 (2.6–3.1)	10		
*p* value					0.940	0.191
**IL-10 (pg/mL)**						
HP	0.2 (0.1–0.4)	8	0.3 (0.2–0.4)	7	−0.0 (−0.5, 0.5)	−0.2 (−1.0–0.5)
LP	0.4 (0.3–0.4)	10	0.3 (0.2–0.5)	10		
*p* value					0.936	0.528
**MCP-1 (pg/mL)**						
HP	325.7 (265.0–347.5)	8	355.4 (305.0–362.8)	7	−22.3 (−58.6, 14.0)	32.8 (−22.0–87.5)
LP	332.8 (239.0–409.2)	10	322.9 (232.7–371.4)	10		
*p* value					0.212	0.223
**Chemerin (ng/mL)**						
HP	208.7 (178.5–219.7)	8	177.9 (155.2–225.9)	7	−20.0 (−35.7, −4.3)	2.1 (−21.6–25.8)
LP	187.8 (147.0–221.4)	10	163.0 (152.8–186.0)	10		
*p* value					0.016	0.854
**Omentin (ng/mL)**						
HP	346.0 (301.0–416.4)	8	333.9 (296.9–405.5)	7	−41.2 (−91.1, 8.7)	21.0 (−54.4–96.4)
LP	384.5 (248.9–491.4)	10	324.1 (221.6–491.3)	10		
*p* value					0.100	0.564
**Leptin (ng/mL)**						
HP	56.8 (43.1–77.9)	8	50.3 (33.0–53.4)	7	−19.2 (−31.9, −6.4)	7.2 (−12.0–26.4)
LP	54.9 (41.8–76.0)	10	36.4 (24.4–45.7)	10		
*p* value					0.006	0.440
**Total adiponectin (µg/mL)**						
HP	5.3 (4.4–7.1)	8	6.4 (5.2–7.1)	7	1.6 (0.3, 2.9)	−1.3 (−3.3–0.6)
LP	4.4 (3.9–6.3)	10	5.6 (4.4–8.9)	10		
*p* value					0.017	0.157
**HMW ^2^ adiponectin (µg/mL)**						
HP	1.3 (0.9–1.9)	5	2.5 (1.6–2.7)	5	0.0 (−0.3, 0.4)	−0.2 (−0.8–0.3)
LP	0.7 (0.4–1.5)	7	1.0 (0.8–2.0)	9		
*p* value					0.855	0.351
**Non-HMW adiponectin (µg/mL)**						
HP	4.7 (3.5–6.9)	8	4.4 (2.3–6.4)	7	1.0 (−0.7, 2.8)	−1.8 (−4.4–0.9)
LP	4.3 (3.7–4.8)	10	4.3 (3.5–8.1)	10		
*p* value					0.236	0.173
**Fetuin-A (µg/mL)**						
HP	254.5 (230.0–300.0)	8	243.0 (194.0–302.0)	7	−13.9 (−44.0, 16.3)	3.2 (−42.3–48.8)
LP	253.0 (228.0–299.0)	10	236.5 (207.0–250.0)	10		
*p* value					0.345	0.882

^1^ All models adjusted for age, sex, body mass index change, baseline biomarker values, and Kenward-Roger (KR) correction, ^2^ high molecular weight; Abbreviations: CI, confidence interval, CRP, C-reactive protein; HMW, high molecular weight; HP, high protein diet; IL-6, interleukin-6; IL-10, interleukin-10; L, liter; LP, low protein diet; MCP-1, monocyte chemoattractant protein 1; mg, milligram; ml, milliliter; *n*, number; ng, nanogram; pg, petagram; TNF-a, tumor necrosis factor alpha; µg, microgram.

## Supplementary Materials

The following are available online at https://www.mdpi.com/2072-6643/12/12/3636/s1, Figure S1: Flow diagram of study population, Table S1: Dietary composition of LP and HP diet groups, Table S2: Spearman partial correlation coefficients and 95% Cis for immune-inflammatory biomarkers, measured at baseline and adjusted for age, sex, and BMI, Table S3: Exemplary food plans for both intervention groups, Table S4: Detection limits (lower level) of ELISA kits per measured biomarker.
