# Supplementary material for: Effects of High and Low Protein Diets on Inflammatory Profiles in People with Morbid Obesity: A 3-Week Intervention Study
Source: Nutrients. 2020 Nov 26;12(12):3636. doi: 10.3390/nu12123636 (PMC7759799; doi:10.3390/nu12123636)
Supplement: Supplementary file 1 [file nutrients-12-03636-s001.pdf]

Supplementary Figure 1. Flow diagram of study population

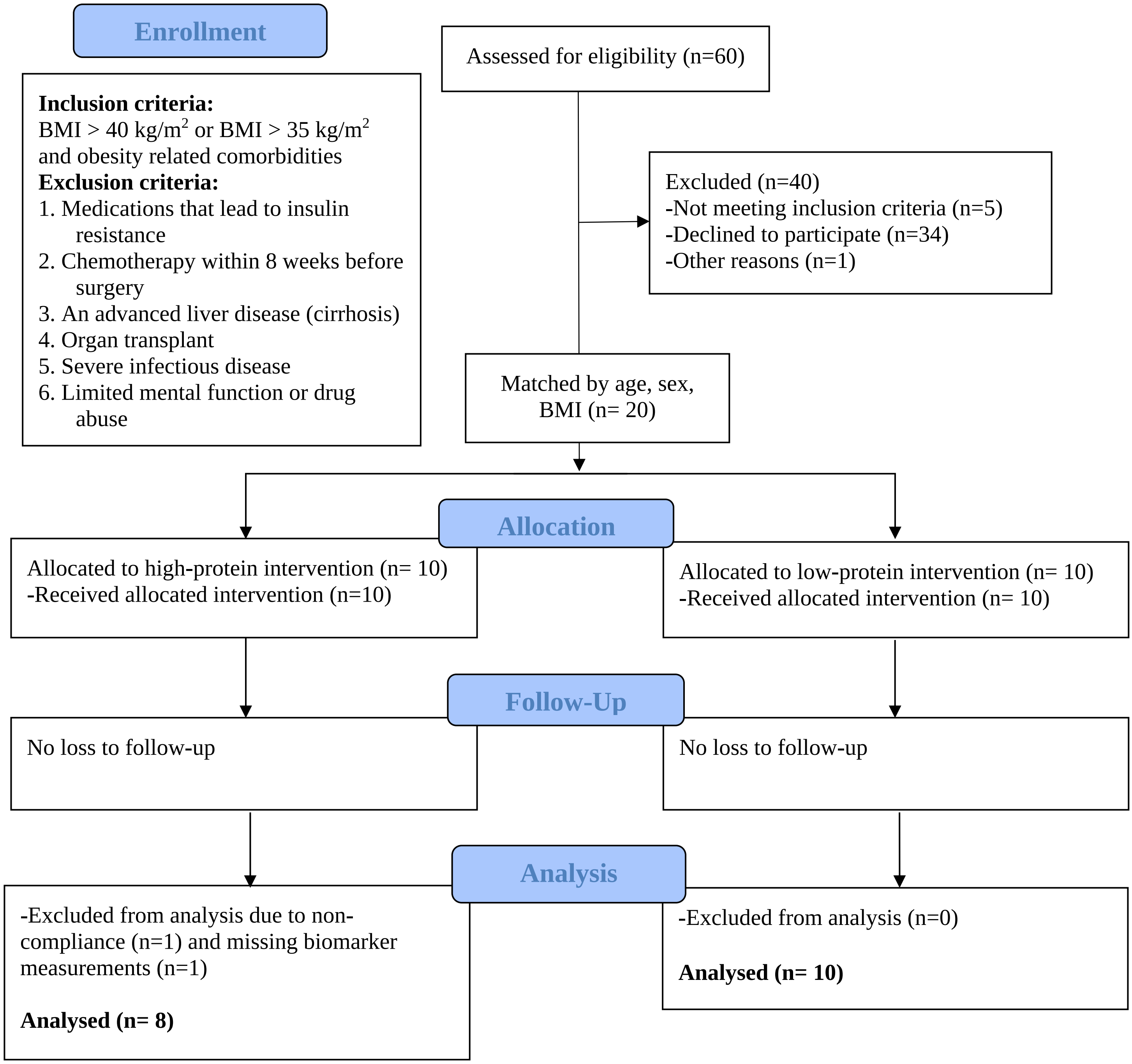

Supplementary Table 1. Nutrient composition of LP and HP diet groups.

		**LP group**	**HP group**	*P*-value*
Energy		1608.9±7.6	1611.1±6.1	0.516
**Protein**	E%	9.5±0.5	32.2±0.7	<.0001
	g	37.5±2.0	126.7±2.5	<.0001
Methionine	mg	483.7±28.4	3074.6±105.4	<.0001
BCAA	mg	5984.4±343.5	24998.3±559.5	<.0001
**Carbohydrates**	E%	58.0±1.1	38.1±1.0	<.0001
	g	229.3±5.3	150.0±3.9	<.0001
Glucose	g	19.2±2.0	11.2±0.8	<.0001
Fructose	g	25.9±3.4	13.4±1.5	<.0001
Saccharose	g	47.6±3.6	23.4±2.6	<.0001
Starch	g	122.2±6.7	61.2±3.0	<.0001
Fibers, soluble	g	10.6±0.5	7.0±0.4	<.0001
Fibers, insoluble	g	24.1±1.4	14.3±0.7	<.0001
**Fat**	E%	28.2±0.9	27.0±1.5	0.047
	g	50.2±1.4	48.0±2.7	0.037
**Fatty acids**				
SFA	g	11.4±1.1	21.2±1.6	<.0001
MUFA	g	19.1±1.1	14.8±0.9	<.0001
PUFA	g	16.0±1.8	7.7±1.1	<.0001

Analysis of dietary plans were performed by PRODI 6.1 expert (Nutriscience, Stuttgart, Germany). Abbreviations: BCAA, branched-chain amino acids leucine, isoleucine, valine; SFA, saturated fatty acids; MUFA, monounsaturated fatty acids; PUFA, polyunsaturated fatty acids.

**P*-values are based on the *t*-test

Supplementary Table 2. Spearman partial correlation coefficients and 95% CIs for immune-inflammatory biomarkers, measured at baseline and adjusted for age, sex, and BMI

	**CRP**		**IL-6**		**TNF-a**		**IL-10**		**MCP-1**		**Chemerin**		**Omentin**		**Leptin**		**Total adiponectin**		**HMW adiponectin**	
	ρ	95% CI	ρ	95% CI	ρ	95% CI	ρ	95% CI	ρ	95% CI	ρ	95% CI	ρ	95% CI	ρ	95% CI	ρ	95% CI	ρ	95% CI
IL-6	0.71	0.28, 0.89																		
P-value	**0.002**																			
TNF-a	-0.07	-0.56, 0.46	0.08	-0.46, 0.56																
P-value	**0.82**		**0.79**																	
IL-10	0.13	-0.41, 0.60	-0.007	-0.52, 0.51	0.14	-0.40, 0.61														
P-value	**0.64**		**0.98**		**0.62**															
MCP-1	-0.11	-0.59, 0.43	0.11	-0.43, 0.59	0.38	-0.17, 0.74	-0.32	-0.70, 0.26												
P-value	**0.70**		**0.69**		**0.16**		**0.27**													
Chemerin	0.09	-0.44, 0.57	0.42	-0.12, 0.76	-0.01	-0.52, 0.50	-0.13	-0.60, 0.41	0.32	-0.24, 0.71										
P-value	**0.75**		**0.11**		**0.96**		**0.66**		**0.26**											
Omentin	-0.06	-0.55, 0.47	0.31	-0.25, 0.71	-0.39	-0.75, 0.16	-0.09	-0.57, 0.44	-0.54	-0.82, -0.02	0.19	-0.36, 0.64								
P-value	**0.84**		**0.26**		**0.15**		**0.75**		**0.04**		**0.50**									
Leptin	0.32	-0.24, 0.71	0.64	0.16, 0.86	0.20	-0.36, 0.64	-0.49	-0.80, 0.05	0.37	-0.19, 0.74	0.44	-0.10, 0.77	0.09	-0.45, 0.57						
P-value	**0.24**		**0.009**		**0.49**		**0.06**		**0.18**		**0.10**		**0.76**							
Total adiponectin	0.13	-0.42, 0.60	0.17	-0.38, 0.62	-0.31	-0.70, 0.25	-0.41	-0.76, 0.14	-0.48	-0.79, 0.06	0.05	-0.48, 0.54	0.46	-0.09, 0.78	0.25	-0.31, 0.67				

P-value	**0.66**		**0.55**		**0.27**		**0.13**		**0.07**		**0.88**		**0.09**		**0.37**					
HMW adiponectin	0.37	-0.41, 0.82	0.52	-0.25, 0.87	-0.53	-0.88, 0.24	-0.48	-0.86, 0.29	-0.27	-0.78, 0.50	0.39	-0.39, 0.83	0.33	-0.44, 0.81	0.45	-0.33, 0.85	0.12	-0.59, 0.72		
P-value	**0.35**		**0.16**		**0.15**		**0.20**		**0.50**		**0.31**		**0.40**		**0.23**		**0.76**			
Fetuin-A	-0.01	-0.52, 0.50	0.08	-0.45, 0.57	0.13	-0.41, 0.60	-0.52	-0.81, 0.005	-0.19	-0.63, 0.37	-0.09	-0.57, 0.44	0.39	-0.17, 0.75	0.21	-0.34, 0.65	0.24	-0.31, 0.67	0.09	-0.61, 0.71
P-value	**0.97**		**0.79**		**0.65**		**0.04**		**0.52**		**0.75**		**0.16**		**0.45**		**0.39**		**0.82**	

Analysis includes all subjects at baseline (n=18). Abbreviations: CI, confidence interval; CRP, C-reactive protein; HMW, high molecular weight; IFNg, interferon gamma, IL, interleukin; MCP1, monocyte chemoattractant protein-1; TNFa, tumor necrosis factor alpha; ρ, Spearman's rank correlation coefficient

Supplementary Table 3. Exemplary food plans for both intervention groups

	Low-protein diet	High-protein diet
**Breakfast**	50 g bread	*Protein shake*
	10 g margarine	30 g protein shake**
	20 g jam	300g 1.5% fat milk
	20 g Nutella	*Toast with jam*
	200 g coffee/tea	30 g whole wheat toast
		5 g margarine
		15 g jam
		200 g coffee/tea
		20 g 1.5% fat milk
**Snack**	100 g raw cucumber	125 g orange without peel
	500 g mineral water	500 g mineral water
**Lunch**	*Pasta with vegetable bolognese*	*Pork steak with potatoes and green beans*
	120 g raw pasta	175 g roast pork steak
	350 g vegetable bolognese	5 g oil
	80 g margarine	150 g potatoes
	500 g mineral water	200 g green beans
		500 g mineral water
**Coffee break**	*Carrot-apple salad*	125 g berries
	80 g raw apple	10 g sugar
	120 g raw carrot	200 g coffee/tea
	2 g lemon juice	20g 1.5% fat milk
	10 g sunflower seed	
	200 g coffee/tea	
**Dinner**	*Bread with avocado*	*Protein shake*

	120 g whole grain bread	30 g protein shake**
	80 g avocado	300g 1.5% fat milk
	50 g tomatoes	*Bread with cheese*
	3 g lemon juice	50 g rye bread
	*Bread with spread**	5 g margarine
	20 g vegan spread	30 g 45% fat Gouda
	80 g raw kiwi	*Mixed salad*
	1000 g mineral water/tea	50 g lettuce
		20 g canned sweetcorn
		100 g cucumber
		80 g tomatoes
		3 g aromatic vinegar
		5 g oil
		1 g herbal salt
		1000 g mineral water/tea

*Vegetarian ‘spreads‘ (Alnatura, Bickenbach, Germany); **Protein shake (WellMix Sport Protein 90, WellMix, Burgwedel, Germany). Ingredients include 42% calcium caseinate, 40% soy protein isolate and 17% whey protein

Supplementary Table 4. Detection limits (lower level) of ELISA kits per measured biomarker

Biomarker	Detection limit
CRP	0.10 mg/L
IL-6	0.33 pg/ml
TNF-a	0.51 pg/ml
IL-10	0.14 pg/ml
MCP-1	0.74 pg/ml
Chemerin	0.1 ng/ml
Omentin	0.5 ng/ml
Leptin	15.6 pg/ml
Total adiponectin	0.47 ng/ml
HMW adiponectin	1.5 ng/ml
Fetuin-A	0.104 ng/ml

Abbreviations: CRP, C-reactive protein; HMW, high molecular weight; IFNg, interferon gamma, IL, interleukin; MCP1, monocyte chemoattractant protein-1; TNFa, tumor necrosis factor alpha
